# Cocaine Use, Unhealthy Alcohol Use, and Pain Interference Among People with HIV

**DOI:** 10.1007/s10461-025-05012-2

**Published:** 2026-01-05

**Authors:** Jonah Sheinin, Thomas D. Brothers, Michael D. Stein, Michael R. Winter, Tibor P. Palfai, Kara M. Magane, Scarlett Bellamy, Mark Asbridge, Theresa W. Kim

**Affiliations:** 1 Dalhousie Medical School, Dalhousie University, 5849 University Avenue, Halifax, NS B3H 4R2 Canada; 2 Division of General Internal Medicine, Department of Medicine, Dalhousie University, Halifax, NS Canada; 3 Addiction Medicine Consult Service, Mental Health & Addictions Program, Nova Scotia Health, Halifax, NS Canada; 4 School of Public Health, Boston University, Boston, MA USA; 5 Department of Psychological and Brain Sciences, Boston University, Boston, MA USA; 6 Department of Epidemiology & Community Health, Dalhousie University, Halifax, NS Canada; 7 Boston Medical Center, Boston University Chobanian & Avedisian School of Medicine, Boston, MA USA

**Keywords:** HIV, Pain, Substance use, Cocaine, Cocaethylene, Alcohol

## Abstract

Pain is prevalent among people with HIV (PWH), and many PWH who experience pain also use substances (illicit drug and/or unhealthy alcohol use). While cocaine use and cocaine and alcohol co-use are prevalent in this population, their effects on pain in PWH are unknown. This study aims to investigate the association of cocaine use and co-use of cocaine and alcohol with pain interference among PWH. We completed a secondary analysis of the Boston Alcohol Research Collaboration on HIV/AIDS (ARCH) study, a longitudinal cohort of PWH with a history of substance use. The outcome was pain interference (Brief Pain Inventory). Exposures were recent cocaine use (Addiction Severity Index) and recent unhealthy alcohol use (Timeline Follow Back). Generalized Estimating Equation (GEE) ordinal logistic regression models were employed, adjusted for demographic factors, illicit/non-medical opioid use and cannabis use. Among 251 participants, 22.3% reported unhealthy alcohol use only, 11.1% reported cocaine use only, and 13.2% reported use of both. Cocaine use was associated with greater pain interference (adjusted odds ratio [aOR]: 1.73, 95% confidence interval [CI]: 1.15–2.60), whether or not participants had unhealthy alcohol use (interaction term, *p* = 0.695). Participants reporting both cocaine and unhealthy alcohol use had greater pain interference than participants reporting neither (aOR: 2.27, 95%CI: 1.35–3.79). Cocaine use was associated with greater pain interference as was co-use of cocaine and unhealthy alcohol among PWH. Considering patterns of substance use can inform clinicians conversations with PWH who may be using substances for pain management.

## Introduction

Pain is a complicating feature of the daily lives of many people with HIV (PWH) [1–3]. Pain may be the result of common co-occurring conditions (e.g., diabetes mellitus, depressive disorders), age-related conditions (e.g., frailty, osteoarthritis), and direct effects of HIV on inflammation, even among those with HIV viral suppression. A disproportionally high prevalence of PWH have chronic pain, often defined as pain that persists beyond three months; estimates range widely between 40% and 53%, and as high as 90% in a cohort study of homeless/marginally housed people with HIV in an urban setting [1–3]. Chronic pain frequently reflects changes in the way that pain signals are generated and interpreted in the body [4, 5]. Pain in PWH often impacts several dimensions of functioning [3]. Pain interference - the degree that the experience of pain limits aspects of daily living, (e.g., physical activity, relationships) - has been linked with a number of quality of life outcomes such as depression, falls, and cognitive decline [6, 7].

Susceptibility to pain and pain thresholds among PWH are also impacted by substances, including illicit drug use, non-medical prescription drug use, and unhealthy quantities or patterns of alcohol use [8, 9]. Substance use can impact the experience of pain in complex and individualized ways. For example, alcohol consumption can have immediate analgesic effects [8, 10–12]. However, prolonged unhealthy alcohol use can also result in hyperalgesia and increased pain interference [10, 12].

Although the association between pain and the use of substances such as opioids and, to a lesser extent, alcohol, has been well characterized, there is comparatively little known about another class of substances, stimulants such as cocaine and amphetamine. This knowledge gap persists despite the increasing use of cocaine and amphetamines and the well-recognized impact of these substances on other important health outcomes (e.g., HIV risk behaviors, overdose) [13, 14]. Stimulant use may also result in hyperalgesia via cocaine and amphetamine-regulated transcript peptide (CARTp), a neuropeptide with pro-nociceptive effects contributing to neuropathic pain [15, 16]. While studies of PWH have reported using cocaine and amphetamines to manage pain, particularly neuropathic pain [11, 17], the impact of stimulants on pain interference has received little examination.

One important complicating factor in determining a possible link between cocaine use and pain interference is comorbid alcohol consumption. A high proportion of people who use cocaine are concurrent drinkers with one meta-analysis estimating the prevalence to be 74% [18]. Those who drink are significantly more likely to use cocaine and vice versa [19]. Beyond the additive effects of alcohol use on pain interference in PWH [20], alcohol may also accentuate the negative effects of cocaine on pain interference. When cocaine and alcohol are used concurrently, cocaine undergoes transesterification to become the metabolite cocaethylene. Studies have shown that cocaethylene produces a more intense, longer euphoria than cocaine alone, and reduces the dysphoria that can be associated with cocaine use, promoting cocaine use and the continued co-use of alcohol [18, 19, 21, 22]. Cocaine and alcohol have neurobiological effects that may interfere with “cognitive adaptations” to pain that are important for reducing distress and physical inactivity, independent of pain severity [23]. Thus, alcohol may have additive and/or synergistic effects with cocaine on pain. We are not aware of research to date that has investigated the effects of concurrent cocaine and alcohol use on pain, especially among PWH.

We examined the association between cocaine use, unhealthy alcohol use, and pain interference among a cohort of PWH testing two hypotheses: (1) cocaine use is associated with greater pain interference; and (2) the use of both cocaine and unhealthy alcohol is associated with greater pain interference than the use of either substance alone.

## Materials and Methods

This is a secondary analysis of the Boston Alcohol Research Collaboration on HIV/AIDS (ARCH) Frailty, Functional impairment, Fractures and Falls (4 F) study, conducted between 2018 and 2022 [24]. Eligibility criteria for the 4 F study included: (1) at least 18 years of age; (2) fluency in English; (3) documentation of HIV infection; and (4) willingness to provide an alternative contact person to assist with study follow-up. In order to be eligible, participants also had to meet at least one of the following substance use-related study entry criteria: (a) any past 12-month use of illicit drugs, cannabis (not recommended by a healthcare provider) or nonmedical use of prescription medications (assessed using the Tobacco, Alcohol, Prescription Medication and Other Substances (TAPS) Tool); (b) past 12-month alcohol use with positive AUDIT-C score (≥ 3 for females and ≥ 4 for males); and/or (c) enrollment in a prior related cohort study of PWH, the entry criterion of which was current substance use disorder or lifetime history of injection drug use [25]. This secondary analysis included all 4 F study participants. There were no additional selection criteria for inclusion in this secondary analysis.

Participants were recruited in-person from an infectious disease and HIV clinic in a large “safety net” urban academic medical center in the northeast United States and enrollment occurred from 2018 to 2020. Participants completed a standardized assessment with trained research staff at baseline and annually thereafter for up to three years.

### Dependent Variable/Primary Outcome

The dependent variable was pain interference, assessed using the Brief Pain Inventory (BPI) [26]. Participants who responded “yes” to the following question were administered the BPI: “Throughout our lives, most of us have had pain from time to time (such as minor headaches, sprains, and toothaches). Have you had pain other than these everyday kinds of pain during the last week?” The BPI Pain interference subscale evaluates the extent to which pain has interfered with domains of their daily life including general activity, mood, walking ability, work, relations with others, sleep, and enjoyment of life over the past seven days [26]. Responses to each question are on a Likert scale (0 = pain does not interfere to 10 = pain interferes completely) and are averaged, yielding an average pain interference score.

Across the whole sample (including people reporting no pain in the past 7 days), we constructed a 4-level ordinal pain interference outcome variable: no pain in the last seven days, and the lowest, middle, and highest tertiles of BPI pain interference scores.

### Independent Variables

Cocaine use was defined as any use in the last 30 days on the Addiction Severity Index (ASI) [27]. Unhealthy alcohol use was defined as any alcohol consumption that exceeded the National Institute on Alcohol Abuse and Alcoholism (NIAAA) recommended weekly or daily limits: >14 drinks in a week or *≥* 5 drinks in a day for males aged 65 years and younger, and > 7 drinks in a week or *≥* 4 drinks in a day for females and males over the age of 65 years. This was assessed with the 14-day alcohol timeline follow back (TLFB) [28, 29]. As such, both cocaine and unhealthy alcohol use were binary (yes/no) variables.

Covariates included demographic variables, age, sex and race/ethnicity, and two time-varying covariates that can impact pain, past 30-day cannabis use and past 30-day illicit/non-prescribed opioid use (ASI). For descriptive purposes, we report the proportion of the sample with HIV viral load > 200 copies, low CD4 count (CD4 < 200) (electronic health record review), and current antiretroviral use (participant self-report).

### Statistical Analyses

Descriptive statistics were generated to describe the baseline characteristics of the sample overall and stratified by cocaine and unhealthy alcohol use. Associations between cocaine use, unhealthy alcohol use, and the 4-category measure of pain interference, (i.e., no pain, tertiles of pain interference score) drew upon repeated cross-sectional analyses, pooling data from all available baseline and annual follow-up visits. All analyses used generalized estimating equations (GEEs), a statistical method that accounts for correlations among repeated measures within the same subjects, enabling valid inference from correlated data. All GEE models were specified with an independence working correlation structure, and results are reported as odds ratios with 95% confidence intervals calculated using robust empirical standard errors.

Two analytic approaches were designed to assess the two study hypotheses : that cocaine use is associated with greater pain interference and that both cocaine and unhealthy alcohol use is associated with even greater pain interference than either substance alone. For the first approach, we fit unadjusted and adjusted GEE ordinal logistic regression models, clustered on participant, to evaluate the association of cocaine use (any vs. none) and the outcome, pain interference. A separate unadjusted GEE ordinal logistic regression model examined the association of unhealthy alcohol use (any vs. none) and pain interference. The adjusted models included cocaine use, unhealthy alcohol use, and adjusted for age, sex and race/ethnicity, past 30-day cannabis use and past 30-day illicit/non-prescribed opioid use. We then assessed whether unhealthy alcohol moderated the association of cocaine use and greater pain interference by adding an interaction term for ‘unhealthy alcohol x cocaine use’ to the multivariable model.

The second analytic approach assessed the two study hypotheses by dividing the sample into mutually exclusive groups: (a) cocaine use and no unhealthy alcohol use, b) unhealthy alcohol use and no cocaine use, c) both cocaine and unhealthy alcohol use (“cocaine/alcohol”), and d) neither cocaine nor unhealthy alcohol use (referent group) For this, we fit unadjusted and adjusted GEE ordinal logistic regression models to evaluate the association of the four-category measure of cocaine and unhealthy alcohol use and pain interference. The same covariates were included in the adjusted model as described in the first analytic approach. The proportional odds assumption for the ordinal logistic regressions was assessed via visual examination of empirical logit plots. All analyses were conducted using SAS 9.4 (SAS Institute, Cary NC).

## Results

### Participant Characteristics

The mean participant age of the study sample (*n* = 251) was of 52.1 years (standard deviation [SD] = 10.5) (Table 1). Nearly one-third of participants were female (32.7%), and the majority were Black non-Hispanic (52.2%). Most were receiving antiretroviral medication and had a HIV viral load < 200, while very few had a CD4 count < 200.

**Table 1:** Baseline characteristics of people with HIV and a history of unhealthy alcohol or other substance use and stratified by recent cocaine and/or unhealthy alcohol use^a^

Characteristic	Total sample *N* = 251	No cocaine or unhealthy alcohol use *N* = 134	Unhealthy alcohol use/no cocaine use *N* = 56	Cocaine use/no unhealthy alcohol use *N* = 28	Cocaine and unhealthy alcohol use *N* = 33
N (% total sample)	251	134 (53.4%)	56 (22.3%)	28 (11.1%)	33 (13.2%)
Age in years, mean (SD)	52.1 (10.5)	52.6 (10.7)	49.7 (11.4)	53.6 (9.2)	52.8 (9.0)
Female	82 (32.7%)	42 (31.3%)	18 (32.1%)	11 (39.3%)	11 (33.3%)
Race/Ethnicity					
Hispanic/Latino	53 (21.1%)	35 (26.1%)	10 (17.9%)	4 (14.3%)	4 (12.1%)
Black, not Hispanic	131 (52.2%)	64 (47.8%)	35 (62.5%)	12 (42.9%)	20 (60.6%)
Multiracial other	20 (8.0%)	9 (6.7%)	3 (5.4%)	4 (14.3%)	4 (12.1%)
White, not Hispanic	47 (18.7%)	26 (19.4%)	8 (14.3%)	8 (28.6%)	5 (15.2%)
HIV viral load ≥ 200	32 (13.3%)	11 (8.5%)	7 (13.0%)	7 (26.9%)	7 (22.6%)
CD4 count < 200	21 (8.6%)	10 (7.6%)	4 (7.4%)	3 (11.1%)	4 (12.9%)
Current antiretroviral therapy use	237 (94.8%)	127 (94.8%)	54 (96.4%)	25 (92.6%)	31 (93.9%)
Cocaine use days, past 30 days					
Mean (SD) Median (IQR)	2.1 (5.7) 0 (0,0)	0 (0) 0 (0,0)	0 (0) 0 (0,0)	8.9 (9.4) 5.5 (2.0,12.5)	8.7 (8.2) 5.0 (3.0,15.0)
Unhealthy drinking days in past 14 days					
Mean (SD) Median (IQR)	1.5 (3.0) 0, (0, 2.0)	0 (0) 0 (0, 0)	4.0 (3.6) 2.0 (1.0, 6.0)	0 (0) 0 (0, 0)	4.8 (3.9) 4.0, (2.0, 6.0)
Cannabis use^b^	125 (49.8%)	60 (44.8%)	34 (60.7%)	11 (39.3%)	20 (60.6%)
Illicit opioid use^b^	40 (15.9%)	11 (8.2%)	6 (10.7%)	14 (50.0%)	9 (27.3%)
Pain interference status					
No pain past 7 days	114 (45.6%)	69 (51.5%)	27 (48.2%)	9 (33.3%)	9 (27.3%)
Lowest tertile score^c^	48 (19.2%)	29 (21.6%)	9 (16.1%)	4 (14.8%)	6 (18.2%)
Middle tertile score^c^	41 (16.4%)	19 (14.2%)	9 (16.1%)	5 (18.5%)	8 (24.2%)
Highest tertile score^c^	47 (18.8%)	17 (12.7%)	11 (19.6%)	9 (33.3%)	10 (30.3%)

SD = Standard Deviation; IQR = Interquartile Range. Percentages are column percentages

^a^Substance use is any illicit drug, prescribed opioid medication misuse, or unhealthy alcohol use

^b^Past 30 days

^c^Past 14 days Brief Pain Inventory Interference Score, higher score indicates more pain interference

Approximately one in five participants (22.3%, 56/251) reported unhealthy alcohol use without cocaine use, and 24.3% reported any cocaine use. Among the latter group, 11.1% (28/251) reported cocaine use without unhealthy alcohol use, and 13.2% (33/251) reported both cocaine and unhealthy alcohol use (Table 1). Almost half (53.4%, 134/251) of the sample reported neither cocaine nor unhealthy alcohol use. The mean number of days with cocaine use were similar among those with cocaine use only compared to those with both cocaine/unhealthy alcohol (mean 8.9 days (SD = 9.4) and 8.7 days (SD = 8.2) of the past 30 days, respectively).

Cannabis use was common in all groups. Illicit/non-medical opioid use varied between cocaine/unhealthy alcohol exposure groups from 8.2% (11/134) of participants with no cocaine/unhealthy alcohol use, 27.3% (9/33) with cocaine/unhealthy use, and 50.0% (14/28) with cocaine use only.

About one-half of the total sample did not report pain (45.6%). Among the participants with pain, the median duration of pain was 24.0 months (interquartile range [IQR]: 1.5, 96.0) and the median pain interference score was 5.6 (IQR: 3.5, 7.5) out of a possible 10 (data not shown).

### Associations Between Cocaine Use, Unhealthy Alcohol Use, and Pain Interference

With the use of GEEs, the models in this paper have a total of *n* = 708 observations. The results of the first analytical approach that assessed the individual associations of recent cocaine use and recent unhealthy alcohol use with pain interference are presented in Table 2. Participants who reported any cocaine use had higher odds of reporting a greater level of pain interference in the unadjusted model (OR = 1.98; 95% CI: 1.37, 2.86), which remained significant in the adjusted model (AOR = 1.73; 95% CI: 1.15, 2.60). Unhealthy alcohol use was not significantly associated with higher pain interference.

**Table 2:** The association of recent cocaine use, recent unhealthy alcohol use and greater pain interference among people with HIV and a history of substance use^a^

Variable	Odds of greater pain interference		
	OR (95% CI)^b^	AOR (95% CI)^c^	AOR (95% CI)^d^
Cocaine use	1.98 (1.37, 2.86)**	1.73 (1.15, 2.60)**	1.60 (0.90, 2.83)
Unhealthy alcohol use	1.28 (0.89, 1.83)	1.26 (0.86, 1.84)	1.21 (0.77, 1.89)
Cocaine use x unhealthy alcohol use	–	–	1.17 (0.53, 2.60)

OR = Odds Ratio; CI = Confidence Interval, AOR = Adjusted Odds Ratio

^a^Results of GEE ordinal logistic regression models using *N* = 708 observations among 251 participants

^b^Separate unadjusted models for cocaine use and unhealthy alcohol use

^c^Results from one model that included cocaine, unhealthy alcohol, age, sex, race/ethnicity, any cannabis use, and any illicit/non-medical opioid use

^d^Results from one model that included cocaine, unhealthy alcohol, age, sex, race/ethnicity, any cannabis use, any illicit/non-medical opioid use, and an interaction term between any cocaine and any unhealthy alcohol use (*p* = 0.695)

*p < 0.05, **p < 0.01

Table 3 presents the results of analyses with the four-category measure of cocaine and unhealthy alcohol exposure. Compared to those who reported no cocaine or unhealthy alcohol use, cocaine/alcohol use was associated with more than double the odds of experiencing greater pain interference (AOR = 2.27; 95% CI: 1.35, 3.79). Cocaine use only was significantly associated with greater pain interference in the unadjusted model only (OR 1.92; 95%CI: 1.15, 3.19), while unhealthy alcohol use only was not significantly associated with greater pain interference in either model.

**Table 3:** Results of unadjusted and adjusted ordinal logistic regression models of the association of a 4-category measure of cocaine and/or unhealthy alcohol use and greater pain interference

	Pain Interference^a^				Odds of greater pain interference^c^	
	No pain	Lowest tertile (0–4.5)	Middle tertile (4.6–6.7)	Highest tertile (6.8–10.0)		
					OR (95% CI)	AOR (95% CI)^c^
Cocaine use only (*n* = 67)^b^	27 (40.3%)	10 (14.9%)	14 (20.9%)	16 (23.9%)	1.92 (1.15, 3.19)*	1.60 (0.90, 2.83)
Unhealthy alcohol only (*n* = 146)^b^	81 (55.5%)	18 (12.3%)	21 (14.4%)	26 (21.9%)	1.09 (0.70, 1.71)	1.21 (0.77, 1.89)
Unhealthy alcohol and cocaine (*n* = 81)^b^	30 (37.0%)	14 (17.3%)	16 (19.75%)	21 (25.9%)	2.12 (1.30, 3.44)**	2.27 (1.35, 3.79)**
No unhealthy alcohol or cocaine (*n* = 414)^b^	229 (55.3%)	69 (16.7%)	60 (14.5%)	56 (13.5%)	REF	REF

OR = Odds Ratio; AOR = Adjusted Odds Ratio

^a^Sample includes participants without pain in the past 7 days. Lowest, middle, highest tertiles refer to Brief Pain Inventory Pain interference scores, which range from 0–10 with higher scores indicating greater pain interference

^b^Row percents

^c^From GEE ordinal logistic regression models including a single 4-category cocaine and unhealthy alcohol exposure variable. *N* = 708 observations. Odds ratios are expressed as odds of higher levels of pain interference

*p < 0.05, **p < 0.01

## Discussion

We investigated the association between recent cocaine use and unhealthy alcohol use with pain interference among people with HIV and a history of substance use. Cocaine was associated with greater pain interference, unhealthy alcohol use alone was not, when compared to persons who used neither substance. The strength of this association was not greater than the effect when considering either substance alone. These results were consistent across two approaches for modelling cocaine and unhealthy alcohol use in participants. It is notable that about half the participants who reported cocaine use also reported unhealthy alcohol use.

This is one of the very few studies examining the association of cocaine and cocaine/alcohol use on pain interference in a sample of persons with HIV infection, despite increasing cocaine use and unhealthy alcohol use in this population. Our finding of an association between cocaine use and greater pain interference among PWH builds on previous research. In qualitative studies by Behar et al. (2020) and Merlin et al. (2015), participants indicated that stimulants have mixed effects when used as analgesics: they may reduce pain in the moment, but the pain may return at a higher level as the high from the substance wears off; or the efficacy of the stimulant may change over time. This may be due to the postulated effect of the ‘cocaine and amphetamine regulated transcript peptide’ (CARTp) [15, 16].

This study’s findings are significant given the gap in research on the growing use of multiple substances [18]. Studies on substance use and pain often do not have detailed information about the substance type, frequency, and recency of use with well-validated instruments. We were able to align the time frame of cocaine and unhealthy alcohol use (past 2 or 4 weeks) with the Brief Pain Inventory (which anchors questions in the past week) and adjust for other types of substance use.

Our study has some limitations. First, it was not possible to discern whether participants who reported both cocaine and alcohol use were using the substances at the same time, making it difficult to assess the impact of cocaethylene versus other potential pathways on pain interference. Second, we examined cross-sectional associations, limiting our capacity to assess the direction of relationship between cocaine and pain interference. We cannot discern if worse pain interference leads to cocaine or cocaine/alcohol use; like the relationship of other substances and pain, these are likely reciprocal relationships. Future research could study longitudinal trajectories. Third, we did not limit the outcome of pain interference to those with chronic pain only, although the duration of pain for most participants exceeded the 3-month threshold for chronic pain. Fourth, we could not completely control for impact that the differential use of opioids and cannabis across the four exposure groups in our study might have on pain interference. Completely controlling for this would require groups that use no other substances beyond cocaine or alcohol, and this would severely limit the sample size. Although we adjusted for cannabis use and opioid use in the analyses, there may therefore still be a confounding effect by these substances. Finally, we were unable to assess potential effects of other stimulant use (i.e., methamphetamines) on pain interference due to the low prevalence of methamphetamine use in this sample and in the study setting (Boston, USA) more generally.

Our findings may help to inform the discussions that healthcare providers have with patients about the impact of stimulant use on pain. Feedback to patients about the potential impact of cocaine on their day-to-day functioning may be valuable, especially for those with chronic pain. Second, it is important to ask patients who are using cocaine about comorbid alcohol use. Avoiding alcohol use when using cocaine may also have some benefit on pain interference, although cocaine use itself risks other negative consequences. Our findings underscore the importance of determining the impact of stimulants on pain-related functioning to support more informed and evidence-based discussions with patients using cocaine for pain self-management.

## Conclusions

Among PWH with a history of substance (illicit drug or unhealthy alcohol) use, recent cocaine use, alone, or with unhealthy levels of alcohol consumption, was associated with greater pain interference. Considering patterns of substance use can inform clinicians conversations with PWH who may be using substances for pain management. Given the prevalence of pain among PWH and the morbidity associated with pain interference, future research could assess whether reductions in cocaine and cocaine/alcohol use may decrease pain interference.
